# The impact of intraarterial, intravenous, and combined tirofiban on endovascular treatment for acute intracranial atherosclerotic occlusion

**DOI:** 10.3389/fneur.2024.1336098

**Published:** 2024-02-13

**Authors:** Zhiping Bu, Dapeng Sun, Gaoting Ma, Baixue Jia, Xu Tong, Xiaochuan Huo, Anxin Wang, Ning Ma, Feng Gao, Dapeng Mo, Ligang Song, Xuan Sun, Yiming Deng, Xiaoqing Li, Bo Wang, Gang Luo, Deguo Su, Zhongrong Miao

**Affiliations:** ^1^Interventional Neuroradiology, Department of Neurology, Beijing Tiantan Hospital, Capital Medical University, Beijing, China; ^2^Department of Neurology, Xuanwu Hospital, Capital Medical University, Beijing, China; ^3^Cerebrovascular Disease Department, Neurological Disease Center, Beijing Anzhen Hospital, Capital Medical University, Beijing, China; ^4^China National Clinical Research Center for Neurological Diseases, Beijing Tiantan Hospital, Capital Medical University, Beijing, China; ^5^Department of Neurology, Liangxiang Hospital of Beijing Fangshan District, Beijing, China

**Keywords:** endovascular treatment, large vessel occlusion, tirofiban, atherosclerotic, mechanical thrombectomy

## Abstract

**Background and purpose:**

Adjunctive tirofiban administration in patients undergoing endovascular treatment (EVT) for acute large vessel occlusion (LVO) has been investigated in several studies. However, the findings are conflict. This study aimed to compare the effect of different administration pathways of tirofiban on patients undergoing EVT for acute LVO with intracranial atherosclerotic disease (ICAD).

**Methods:**

Patients were selected from the ANGEL-ACT Registry (Endovascular Treatment Key Technique and Emergency Workflow Improvement of Acute Ischemic Stroke: A Prospective Multicenter Registry Study) and divided into four groups: intra-arterial (IA), intravenous (IV), and intra-arterial plus intravenous (IA+IV) and non-tirofiban. The primary outcome was 90-day ordinal modified Rankin Scale (mRS) score, and the secondary outcomes included the rates of mRS 0–1, 0–2, and 0–3 at 90-day, successful recanalization. The safety outcomes were symptomatic intracranial hemorrhage (sICH) and other safety endpoints. The multivariable logistic regression models adjusting for potential baseline confounders were performed to compare the outcomes. A propensity score matching (PSM) with a 1:1:1:1 ratio was conducted among four groups, and the outcomes were then compared in the post-matched population.

**Results:**

A total of 502 patients were included, 80 of which were in the IA-tirofiban group, 73 in IV-tirofiban, 181 in (IA+IV)-tirofiban group, and 168 in the non-tirofiban group. The median (IQR) 90-day mRS score in the four groups of IA, IV, IA+IV, and non-tirofiban was, respectively 3(0–5) vs. 1(0–4) vs. 1(0–4) vs. 3(0–5). The adjusted common odds ratio (OR) for 90-day ordinal modified Rankin Scale distribution with IA-tirofiban vs. non-tirofiban was 0.77 (95% CI, 0.45–1.30, *P* = 0.330), with IV-tirofiban vs. non-tirofiban was 1.36 (95% CI, 0.78–2.36, *P* = 0.276), and with (IA+IV)-tirofiban vs. non-tirofiban was 1.03 (95% CI, 0.64–1.64, *P* = 0.912). The adjusted OR for mRS 0–1 and mRS 0–2 at 90-day with IA-tirofiban vs. non-tirofiban was, respectively 0.51 (95% CI, 0.27–0.98, *P* = 0.042) and 0.50 (95% CI, 0.26–0.94, *P* = 0.033). The other outcomes of each group were similar with non-tirofiban group, all *P* was >0.05. After PSM, the common odds ratio (OR) for 90-day ordinal modified Rankin Scale distribution with IA-tirofiban vs. non-tirofiban was 0.41 (95% CI, 0.18–0.94, *P* = 0.036), and the OR for mRS 0–1 and mRS 0–2 at 90-day with IA-tirofiban vs. non-tirofiban was, respectively 0.28 (95% CI, 0.11–0.74, *P* = 0.011) and 0.25 (95% CI, 0.09–0.67, *P* = 0.006).

**Conclusions:**

Intra-arterial administration of tirofiban was associated with worse outcome than non-tirofiban, which suggested that intra-arterial tirofiban had a harmful effect on patients undergoing EVT for ICAD-LVO.

**Clinical trial registration:**

http://www.clinicaltrials.gov, Unique identifier: NCT03370939.

## Introduction

The benefit of tirofiban administration in patients with AIS undergoing mechanical thrombectomy is still unknow. The recently published randomized RESCUE BT (The Endovascular Treatment With vs. Without Tirofiban for Patients with Large Vessel Occlusion Stroke) trial explored the safety and efficacy of intravenous tirofiban in patients with acute anterior circulation LVO, this study didn't find that tirofiban could improve significantly outcomes compared with placebo, but suggested intravenous tirofiban could benefit the patients with large artery atherosclerosis (LAA) in subgroup analysis (1). Furthermore, in the *post-hoc* analysis of RESCUE BT trial, it was found that intravenous tirofiban was an effective adjunctive medication for patients with ICAD- related LVO undergoing EVT (2). In addition, it was reported that patients treated with intra-arterial tirofiban undergoing EVT for AIS suffered from an increased risk of symptomatic and fatal intracerebral hemorrhage (3). A recent meta-analysis also reported that treatment with tirofiban in patients with AIS undergoing EVT was effective in improving prognosis, particularly in patients with large atherosclerotic stroke, and intravenous administration of tirofiban improved more significantly clinical prognosis of patients than arterial administration (4). In practice, the dose and administration pathways of tirofiban are decided by interventionists at discretion, but the dose and pathway of tirofiban in the RESCUE BT trial were restricted, so whether the result of the RESCUE BT trial can be generalized to a broader population and clinical setting is still needed to be explored. Therefore, in this study, we aimed to explore the efficacy and the safety of different administration pathways of tirofiban on patients undergoing EVT for acute ICAD-related LVO based on the data of the ANGEL-ACT (a prospective nationwide registry study).

## Methods

### Study population

The patients were selected from the ANGEL-ACT (endovascular treatment key technique and emergency workflow improvement of acute ischemic stroke) registry, which was a nationwide prospective cohort including 1,793 consecutive adult patients with AIS undergoing EVT for LVO at 111 hospitals from 26 provinces in China between November 2017 and March 2019. The inclusion/exclusion criteria, data collection and methods of the ANGEL-ACT registry were reported in previous article (5). The exclusion criteria of this analysis were as follows: (1) EVT records unavailable; (2) No underlying ICAD after reopening of the occluded vessel or underlying ICAD could not be evaluated according to our criteria; (3) 90-day mRS missing; (4) Only intravenous bolus injection of tirofiban. Finally, we included 502 eligible patients in this analysis. The study protocol of ANGEL-ACT registry was approved by the Ethics Committees of Beijing Tiantan Hospital, Capital Medical University, and all participating centers, written informed consent were provided by the patients or their legally authorized representatives before the study enrollment.

Finally, 502 patients were divided into four groups. One hundred and sixty-two patients who did not receive any tirofiban treatment were assigned to the control (non-tirofiban) group, 80 patients who only received intraarterial tirofiban were assigned to the IA-tirofiban group, 73 patients who only received intravenous tirofiban were assigned to the IV-tirofiban group, and 181 patients who received both intraarterial and intravenous tirofiban were assigned to the (IA+IV)-tirofiban group.

### Data collection

All information including demographic characteristics, vascular risk factors, physical examination findings, neurovascular images, preprocedural treatment, time-metric data, procedural details, periprocedural management, any adverse events within 90 days and modified Rankin Scale (mRS) (6) at 90 days were prospectively collected. All investigators who recorded the National Institutes of Health Stroke Scale (NIHSS) and mRS were trained and competent. All images including baseline computed tomography (CT)/magnetic resonance (MR), computed tomography angiography (CTA)/magnetic resonance angiography (MRA), digital subtraction angiography (DSA), and follow-up CT/MRI were evaluated by an imaging core laboratory which was blinded to clinical data and outcomes. Related diagnostic analyses of all images were independently done by two neuroradiologists, with a third available if needed to be adjudicated. The follow-up CT or MRI was performed immediately and 24 ± 2 h after the procedure, and intracranial hemorrhage (ICH) was assessed based on the post-procedural imaging. Early ischemic changes on CT were identified by using the Alberta Stroke Program Early CT Score (ASPECTS) for anterior circulation stroke (7), and the posterior circulation (PC)–ASPECTS for posterior circulation stroke (8) based on baseline CT. The variable of admission ASPECTS in this article referred to ASPECTS or pc-ASPECTS. Assessments of occlusion site, presence of tandem extracranial stenosis/occlusion, intraprocedural embolization, and modified thrombolysis in cerebral ischemia score (mTICI) (9) were based on the DSA.

### Intervention of tirofiban

The decision of tirofiban treatment and administration pathway was at the discretion of interventionists. In general, tirofiban was administrated when interventionists encountered the following conditions: (1) Emergency stenting or balloon angioplasty for severe residual stenosis or instant re-occlusion; (2) Severe atherosclerosis disease in occlusive site with a high risk of early re-occlusion; (3) Successful mechanical recanalization with three or more passes with a stent retriever for presumed endothelial damage. The regimen of administration was not mandatory. It was generally recommended that, if necessary, tirofiban should be applied after recanalization, with local arterial administration through the guiding catheter. A low-dose intra-arterial bolus (400– 500 μg) followed by a continuous intravenous infusion (300–480 μg/h) for 24 h was proposed as a standard administration and at 4 h prior to the end of the infusion, dual antiplatelet agents (aspirin 100 mg and clopidogrel 75 mg) were taken orally if ICH was excluded by computed tomography or magnetic resonance imaging.

### Definition of ICAD-LVO

The ICAD-LVO was defined as occlusion located in the intracranial artery and was caused by acute *in situ* thrombus secondary to underlying ICAD. Underlying ICAD was defined as fixed residual stenosis degree >50% or stenosis with distal blood flow impairment or evidence of repeated re-occlusion, can also be determined according to previous images indicating stenotic lesion at the occlusion, at the same time vasospasm, dissection, vasculitis, or Moyamoya disease were excluded (10, 11), and was diagnosed by the imaging core laboratory.

### Outcomes measurement

The primary outcome was the 90-day ordinal mRS with scores ranging from 0 (no symptoms) to 6 (death). The secondary outcomes included the rates of mRS 0–1, 0–2, and 0–3 at 90 days, changes in the NIHSS score at 24 h and 7 days from baseline as assessed, rates of successful recanalization at final angiogram defined as modified Thrombolysis in Cerebral Infarction (mTICI) of 2b to 3 and complete recanalization defined mTICI of 3, and the pass numbers of thrombectomy. The safety outcomes were any ICH and symptomatic ICH within postprocedural 24 h according to the Heidelberg Bleeding Classification (12), intraprocedural embolization in procedure, and death within 90 days.

### Statistical analysis

Continuous variables were presented as the median [interquartile range (IQR)] and categorical variables as a number (percentage). Comparisons of the baseline, procedural characteristics and outcomes among four groups were performed using the Kruskal–Wallis test for continuous and Pearson χ^2^-test for categorical variables. In the univariable analysis, the *P*-value < 0.05 indicated that variables were not entirely equal in the four groups and served as confounder. we performed ordinal/binary logistic regression or generalized linear models by adjusting for confounders to calculate the common odds ratios (OR), OR or β-coefficients with 95% confidence intervals (CI), comparing the clinical outcomes of the three groups of tirofiban with non-tirofiban. To reduce selection bias, a propensity score matching (PSM) was performed among the four groups. All variables with *P* < 0.05 in the univariable analysis were included to generate the propensity score. We matched four groups using a greedy-matching algorithm without replacement at a 1:1:1:1 ratio, with a caliper width ≤ 0.2 of the standard deviation of the logit of the propensity scores. Finally, we explored whether the effects of different administration Pathways of tirofiban on the primary outcome differed in certain subgroups by testing the administration pathway of tirofiban by subgroup interaction effect using an ordinal logistic regression model in the following subgroups: gender (female vs. male), age (age<65 vs. age ≥ 65), baseline NIHSS (<15 vs. ≥15), baseline ASPECTS (≤9 vs. =10), occlusion location (posterior circulation vs. anterior circulation), tandem lesions (yes vs. no), onset-to-puncture time (<6 vs. ≥6 h), pretreatment with IVT (yes vs. no), rescue balloon/stenting angioplasty (yes vs. no). Significance level was set to P = 0.05 (2-sided). We used SAS software v.9.4 (SAS Institute, Cary, NC, USA) to conduct the statistical analyses.

## Results

We calculated that in 261 patients receiving IA-tirofiban, the median bolus dose of IA-tirofiban was 5.7 μg/kg, the interquartile range was 4.3–7.6 μg/kg and in 254 patients receiving IV-tirofiban, median intravenous infusion speed was 0.07 μg/kg/min, the interquartile range was 0.06–0.08 μg/kg/min. Finally, 168 patients were assigned to non-tirofiban group, 80 people were assigned to the IA-tirofiban group, 73 patients were assigned to the IV-tirofiban group, and 181 patients were assigned to the (IA+IV)-tirofiban group.

### Baseline characteristics

**Figure 1:**
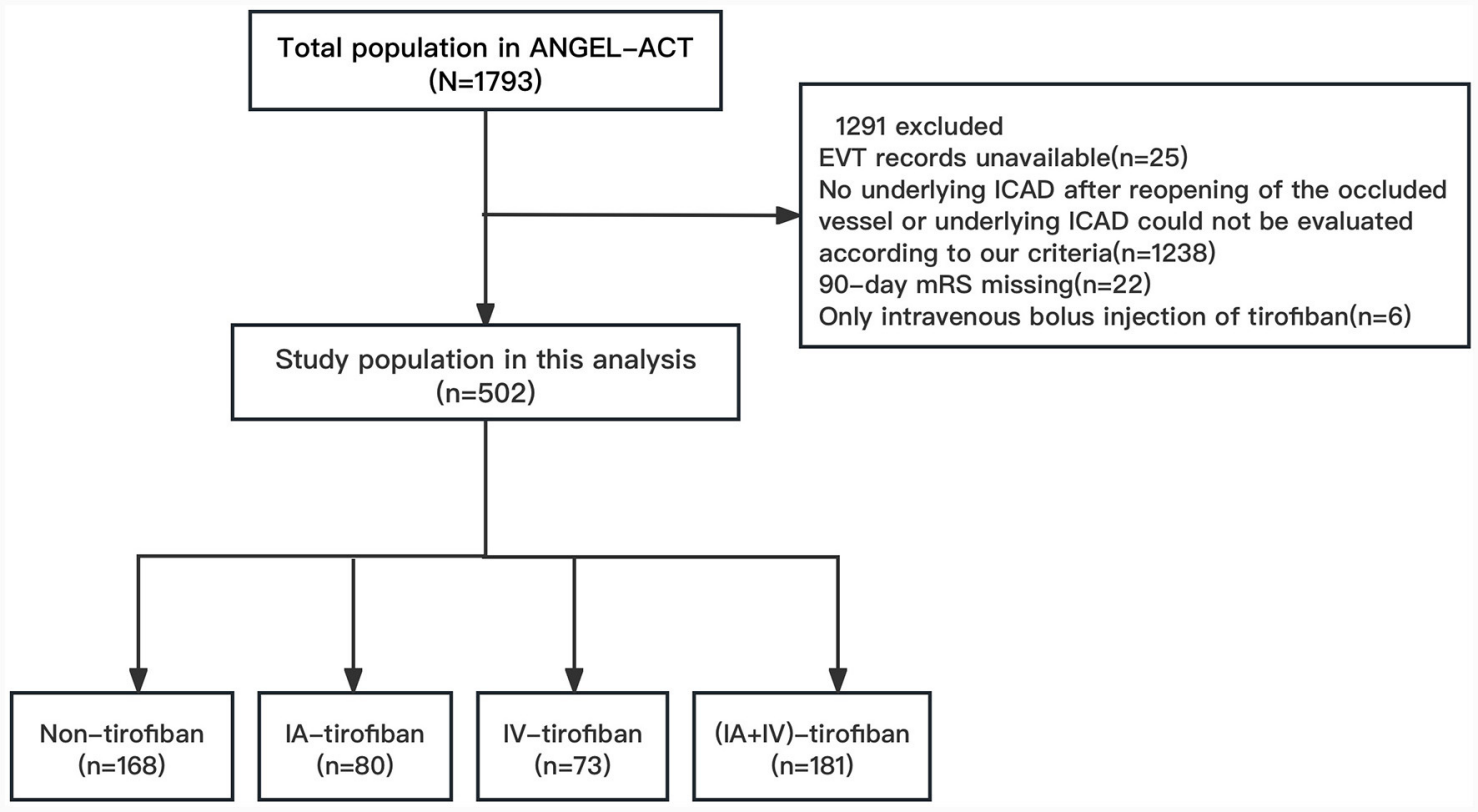
Flowchart of patient selection. EVT, endovascular treatment; ICAD, Intracranial atherosclerotic disease; mRS, modified Rankin Scale; IA, intra-arterial; IV, intravenous.

We enrolled 1,793 AIS patients who underwent EVT, 502 of which were included in this study based on the exclusion criteria (Figure 1). The baseline clinical and procedural characteristics of the eligible patients were presented in Table 1. The proportions of men in non-tirofiban, IA-tirofiban, IV-tirofiban, and (IA+IV)-tirofiban groups were, respectively 70.2 vs. 82.5 vs. 76.7 vs. 84.0%, and the proportion in (IA+IV)-tirofiban group was higher compared to the other three groups (*P* = 0.013). The median (IQR) of ages in non-tirofiban, IA-tirofiban, IV-tirofiban and (IA+IV)-tirofiban groups were respectively 65 (55–71) vs. 61 (53.5–69) vs. 61 (52–68) vs. 61 (54–68), the difference among four groups was not statistically significant (*P* = 0.088). (IA+IV)-tirofiban group had higher prevalence of hypertension (76.2 vs. 58.9 vs. 61.3 vs. 58.9%, *P* = 0.002) and hyperlipidemia (18.8 vs. 8.9 vs. 5.0 vs. 8.2%, *P* = 0.003), less frequency of intra-arterial thrombolysis (2.8 vs. 14.3 vs. 10.0 vs. 9.6%, *P* = 0.002) and higher rate of rescue balloon/stenting angioplasty (77.9 vs. 40.5 vs. 50.0 vs. 53.4%, *P* < 0.001) compared to Non-tirofiban, IA-tirofiban, and IV-tirofiban groups. Non-tirofiban group had higher prevalence of atrial fibrillation (19.6 vs. 8.8 vs. 6.9 vs. 2.2%, *P* < 0.001) and more frequency of prior use of antiplatelet agents (24.4 vs. 13.8 vs. 13.7 vs. 13.3%, *P* = 0.024) compared to IA-tirofiban and IV-tirofiban and (IA+IV)-tirofiban groups (Table 1).

**Table 1:** Baseline characteristics of different tirofiban groups.

**Variables**	**Total (*n* = 502)**	**Non-tirofiban (*n* = 168)**	**IA-tirofiban (*n* = 80)**	**IV-tirofiban (*n* = 73)**	**(IA+IV)- tirofiban (*n* = 181)**	***P*-value**
**Baseline characteristics**						
Male, *n* (%)	392 (78.1)	118 (70.2)	66 (82.5)	56 (76.7)	152 (84.0)	0.013
Age, y, median (IQR)	63 (54–69)	65 (55–71)	61 (53.5–69)	61 (52–68)	61 (54–68)	0.088
Hypertension, *n* (%)	329 (65.5)	99 (58.9)	49 (61.3)	43 (58.9)	138 (76.2)	0.002
Diabetes, *n* (%)	109 (21.7)	30 (17.9)	24 (30.0)	11 (15.1)	44 (24.3)	0.063
Hyperlipidemia, *n* (%)	59 (11.8)	15 (8.9)	4 (5.0)	6 (8.2)	34 (18.8)	0.003
Coronary heart disease, *n* (%)	53 (10.6)	20 (11.9)	9 (11.3)	10 (13.7)	14 (7.7)	0.448
Atrial fibrillation, *n* (%)	49 (9.8)	33 (19.6)	7 (8.8)	5 (6.9)	4 (2.2)	< 0.001
Prior stroke, *n* (%)	128 (25.5)	43 (25.6)	12 (15.0)	18 (24.7)	55 (30.4)	0.074
**Smoking history**, ***n*** **(%)**						
Never smoking	253 (50.4)	98 (58.3)	46 (57.5)	32 (43.8)	77 (42.5)	0.012
Current smoking	219 (43.6)	60 (35.7)	30 (37.5)	33 (45.2)	96 (53.0)	
Previous smoking	30 (6.0)	10 (6.0)	4 (5.0)	8 (11.0)	8 (4.4)	
SBP, mmHg, median (IQR)	148.5 (134–165)	145 (130–160)	146 (132.5–167)	145 (137–160)	150 (140–170)	0.022
Admission NIHSS, median (IQR)^§^	15 (10.5–21.5)	16 (11–21)	17 (11.5–23)	15 (11–19)	14.5 (10–20)	0.349
Admission ASPECTS, median (IQR)^#^	8 (7–10)	9 (7–10)	9 (7–10)	9 (6–10)	8 (6–10)	0.001
Anterior circulation	338 (67.3)	119 (70.8)	50 (62.5)	56 (76.7)	113 (62.4)	0.083
Posterior circulation	164 (32.7)	49 (29.2)	30 (37.5)	17 (23.3)	68 (37.6)	0.083
**Occlusion sites**						
ICA	99 (19.7)	44 (26.2)	11 (13.8)	13 (17.8)	31 (17.1)	0.037
M1	211 (42.0)	62 (36.9)	33 (41.3)	41 (56.2)	75 (41.4)	
VBA	161 (32.1)	48 (28.6)	30 (37.5)	17 (23.3)	66 (34.5)	
Other 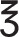	31 (6.2)	14 (8.3)	6 (7.5)	2 (2.7)	9 (5.0)	
Tandem lesions	108 (21.5)	39 (23.2)	18 (22.5)	16 (21.9)	35 (19.3)	0.838
OTD time, median (IQR), min 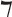	176 (70–318.5)	142.5 (50–260)	227 (120–450)	200 (96.5–330)	177 (80–360)	0.004
DTP time, median (IQR), min^¶^	122.5 (85–188.5)	119.5 (80–184)	129.5 (80.5–224)	200 (96.5–330)	130 (95–209)	0.041
PTR time, median (IQR), min^†^	99 (60–147)	98 (60–139)	103.5 (69–169.5)	98 (60–144)	99 (58–150)	0.757
OTR time, median (IQR), min^‡^	457.5 (340–659)	406 (315–556)	532.5 (431–750)	468 (340–630)	468 (341–708.5)	< 0.001
OTP time, median (IQR), min 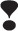	345 (226–525)	291 (200–428)	393.5 (264–660)	338 (231–495)	351.5 (226.5–585)	0.002
General anesthesia, *n* (%)	215 (42.8)	74 (44.1)	32 (40.0)	30 (41.1)	79 (43.7)	0.918
Prior use of antiplatelet agents	86 (17.1)	41 (24.4)	11 (13.8)	10 (13.7)	24 (13.3)	0.024
Prior use of anticoagulants	9 (1.8)	3 (1.8)	2 (2.5)	2 (2.7)	2 (1.1)	0.781
Prior IVT, *n* (%)	123 (24.5)	41 (24.4)	25 (31.3)	15 (20.6)	42 (23.2)	0.432
Heparin, *n* (%)	253 (50.4)	94 (56.0)	40 (50.0)	32 (43.8)	87 (48.1)	0.292
IAT, *n* (%)	44 (8.8)	24 (14.3)	8 (10.0)	7 (9.6)	5 (2.8)	0.002
Stent retriever as first-line, *n* (%)	318 (63.4)	99 (58.9)	54 (67.5)	42 (57.5)	123 (68.0)	0.193
Direct aspiration as first-line, *n* (%)	20 (4.0)	8 (4.8)	5 (6.3)	1 (1.4)	6 (3.3)	0.414
Direct aspiration+ stent retriever as first-line	40 (8.0)	16 (9.5)	3 (3.8)	9 (12.3)	12 (6.6)	0.185
Rescue balloon/stenting angioplasty, *n* (%)	288 (57.4)	68 (40.5)	40 (50.0)	39 (53.4)	141 (77.9)	< 0.001

IA, intra-arterial; IV, intravenous; SBP, systolic blood pressure; IQR, interquartile range; NIHSS, National Institutes of Health Stroke Scale; ASPECTS, Alberta Stroke Program Early CT Score; ICA, internal carotid artery; M1, middle cerebral artery M1 segment; VBA, Vertebrobasilar artery; OTD, onset-to-door; DTP, door to-puncture; PTR, puncture-to-recanalization; OTR, onset-to-recanalization; OTP, onset-to-puncture; IVT, intravenous thrombolysis; IAT, intra-arterial thrombolysis.

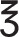
Other including middle cerebral artery M2 segment, anterior cerebral artery A1/A2 segments and posterior cerebral artery P1 segment.

§ 2 missing data.

# 2 missing data, ASPECTS for anterior circulation stroke and pc-ASPECTS for posterior circulation stroke.

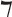
14 missing data.

¶ 46 missing data.

† 1 missing data.

‡ 4 missing data

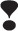
4 missing data.

### Outcomes

**Figure 2:**
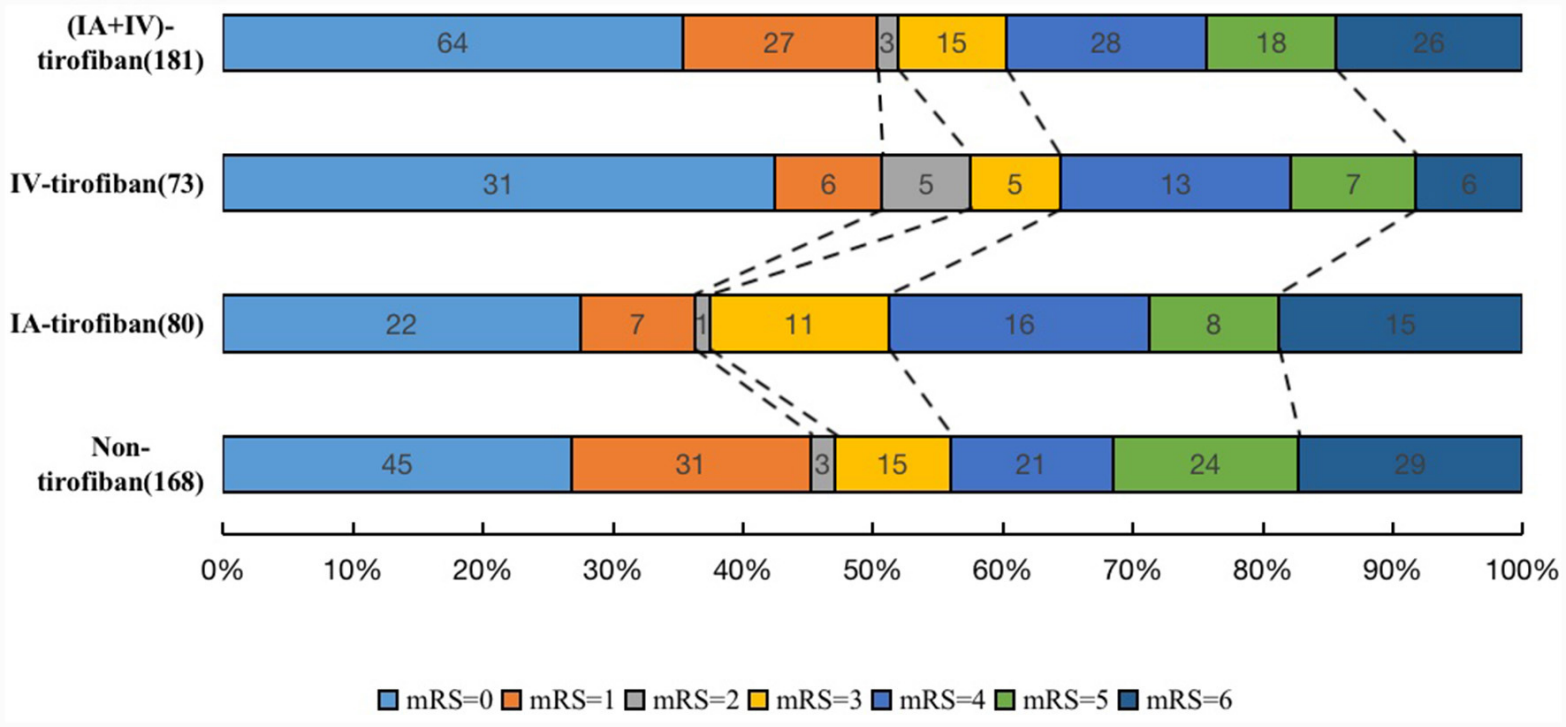
The shift on the 90-d modified Rankin Scale (mRS) score in patients of non-tirofiban, intra-arterial (IA) tirofiban, intravenous (IV) tirofiban, and intra-arterial plus intravenous (IA+IV) tirofiban.

The median (IQR) 90-day mRS score in the four groups of IA, IV, IA+IV, and non-tirofiban was, respectively 3 (0–5) vs. 1 (0–4) vs. 1 (0–4) vs. 3 (0–5). After adjusting the cofounders including male, hypertension, hyperlipidemia, atrial fibrillation, smoking history, admission systolic blood pressure, admission ASPECTS, occlusion sites, onset-to-door time, door-to-puncture time, onset-to-recanalization time, onset-to-puncture time, prior use of antiplatelet agents, IAT and Rescue balloon/stenting angioplasty, the common OR for 90-day ordinal modified Rankin Scale distribution with IA-tirofiban vs. non-tirofiban was 0.77 (95% CI, 0.45–1.30, *P* = 0.330), with IV-tirofiban vs. non-tirofiban was 1.36 (95% CI, 0.78–2.36, *P* = 0.276), and with (IA+IV)-tirofiban vs. non-tirofiban was 1.03 (95% CI, 0.64–1.64, *P* = 0.912). The shift on the 90-d mRS score in patients of non-tirofiban, IA-tirofiban, IV-tirofiban, and (IA+IV)-tirofiban groups was depicted in Figure 2. The rates of mRS 0–1 in IA-tirofiban and non-tirofiban groups were respectively 36.3 vs. 45.2%, after adjusting the cofounders the OR was 0.51 (95% CI, 0.27–0.98, *P* = 0.042). The rates of mRS 0 to 2 in IA-tirofiban and non-tirofiban groups were respectively 37.5 vs. 47.0%, after adjusting the cofounders the OR was 0.50 (95% CI, 0.26–0.94, *P* = 0.033). The other outcomes of each group were similar with non-tirofiban group, all *P* was >0.05 after adjusting the cofounders (Tables 2, 3).

**Table 2:** Outcomes of different tirofiban groups.

**Variables**	**Total (*n* = 502)**	**Non-tirofiban (*n* = 168)**	**IA-tirofiban (*n* = 80)**	**IV-tirofiban (*n* = 73)**	**(IA+IV)- tirofiban (*n* = 181)**	***P*-value**
mRS at 90 d, median (IQR)	3 (0–5)	3 (0–5)	3 (0–5)	1 (0–4)	1 (0–4)	0.057
mRS 0–1 at 90 d, *n* (%)	233 (46.4)	76 (45.2)	29 (36.3)	37 (50.7)	91 (50.3)	0.169
mRS 0–2 at 90 d, *n* (%)	245 (48.8)	79 (47.0)	30 (37.5)	42 (57.5)	94 (51.9)	0.065
mRS 0–3 at 90 d, *n* (%)	291 (58.0)	94 (56.0)	41 (51.3)	47 (64.4)	109 (60.2)	0.338
Change in NIHSS score at 24 h, median (IQR) 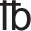	−4.0 (−9 to 0)	−4 (−8 to −1)	−4 (−8 to 0)	−4 (−9.5 to −1.5)	−5 (−10 to 0)	0.341
Change in NIHSS score at 7 d, median (IQR) 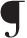	−7 (−12 to −2)	−6 (−12 to −2)	−6 (−13 to −1)	−8 (−11 to −3)	−7 (−12.5 to −2)	0.766
Complete recanalization, *n* (%)	296 (59.0)	94 (56.0)	36 (45.0)	44 (60.3)	122 (67.4)	0.006
Successful recanalization, *n* (%)	464 (92.4)	154 (91.7)	73 (91.3)	64 (87.7)	173 (95.6)	0.156
Pass number of thrombectomy, median (IQR)	1 (1–2)	1 (1–2)	2 (1–3)	1 (1–2)	1 (1–2)	0.265
Symptomatic ICH within 24 h *n* (%)^?!^	19 (3.9)	8 (4.9)	1 (1.3)	5 (6.9)	5 (2.9)	0.253
Any ICH within 24 h, *n* (%)^??^	79 (16.2)	30 (18.2)	13 (16.9)	9 (12.5)	27 (15.4)	0.727
Death within 90 d, *n* (%)	76 (15.1)	29 (17.3)	15 (18.8)	6 (8.2)	26 (14.4)	0.240
Intraprocedural embolization, *n* (%)	19 (3.8)	7 (4.2)	2 (2.5)	2 (2.7)	8 (4.4)	0.838

IA, intra-arterial; IV, intravenous; mRS modified Rankin Scale; IQR, interquartile range; NIHSS, National Institutes of Health Stroke Scale; ICH, intracranial hemorrhage.

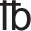
 31 missing data.

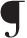
 39 missing data.

^??^13 missing data.

^?!^15 missing data.

**Table 3:** Common OR or OR of safety and efficacy outcome according to different regimen of tirofiban.

**Outcomes variables**	**IA-tirofiban vs. non-tirofiban**				**IV-tirofiban vs. non-tirofiban**				**(IA**+**IV)- tirofiban vs. non-tirofiban**			
	**Unadjusted effect size (95% CI)**	***P*** **-value**	**Adjusted** ^*^ **effect size (95% CI)**	***P*** **-value**	**Unadjusted effect size (95% CI)**	***P*** **-value**	**Adjusted** ^*^ **effect size (95% CI)**	***P*** **-value**	**Unadjusted effect size (95% CI)**	***P*** **-value**	**Adjusted** ^*^ **effect size (95% CI)**	***P*** **-value**
mRS at 90 d, median (IQR)	0.91 (0.57 to 1.45)	0.690	0.77 (0.45 to 1.30)	0.330	1.74 (1.07 to 2.86)	0.027	1.36 (0.78 to 2.36)	0.276	1.33 (0.92 to 1.94)	0.129	1.03 (0.64 to 1.64)	0.912
mRS 0–1 at 90 d, *n* (%)	0.69 (0.40 to 1.19)	0.182	0.51 (0.27 to 0.98)	0.042	1.24 (0.72 to 2.16)	0.437	0.99 (0.52 to 1.87)	0.970	1.22 (0.80 to 1.87)	0.347	1.02 (0.59 to 1.75)	0.956
mRS 0–2 at 90 d, *n* (%)	0.68 (0.39 to 1.17)	0.159	0.50 (0.26 to 0.94)	0.033	1.53 (0.88 to 2.66)	0.135	1.11 (0.59 to 2.10)	0.750	1.22 (0.80 to 1.85)	0.360	0.88 (0.51 to 1.53)	0.659
mRS 0–3 at 90 d, *n* (%)	0.83 (0.49 to 1.41)	0.487	0.75 (0.40 to 1.39)	0.357	1.42 (0.81 to 2.51)	0.223	1.07 (0.55 to 2.05)	0.850	1.19 (0.78 to 1.83)	0.420	0.97 (0.56 to 1.70)	0.925
Change in NIHSS score at 24 h, median (IQR) 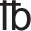	0.90 (−1.15 to −2.94)	0.389	0.94 (−1.42 to 3.29)	0.436	0.73 (−1.79 to 3.26)	0.568	1.02 (−1.69 to 3.74)	0.460	0.26 (−2.38 to 2.90)	0.847	0.40 (−2.40 to 3.20)	0.779
Change in NIHSS score at 7 d, median (IQR) 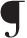	0.86 (−1.32 to 3.04)	0.440	1.56 (−0.88 to 3.99)	0.210	0.38 (−2.34 to 3.10)	0.782	0.62 (−2.21 to 3.44)	0.668	−0.67 (−3.50 to 2.16)	0.643	−0.06 (−2.96 to 2.84)	0.966
Complete recanalization, *n* (%)	0.64 (0.38 to 1.10)	0.107	0.63 (0.34 to 1.18)	0.151	1.19 (0.68 to 2.09)	0.533	1.09 (0.58 to 2.06)	0.796	1.63 (1.05 to 2.52)	0.028	1.50 (0.86 to 2.61)	0.151
Successful recanalization, *n* (%)	0.95 (0.37 to 2.45)	0.912	0.94 (0.31 to 2.85)	0.908	0.65 (0.27 to 1.57)	0.335	0.36 (0.12 to 1.06)	0.065	1.97 (0.80 to 4.81)	0.139	1.08 (0.35 to 3.38)	0.889
Pass number of thrombectomy, median (IQR)	0.03 (−0.25 to 0.32)	0.820	−0.11 (−0.45 to 0.23)	0.530	0.32 (−0.04 to 0.69)	0.078	0.29 (−0.10 to 0.68)	0.149	−0.17 (−0.54 to 0.20)	0.371	−0.25 (−0.65 to 0.14)	0.210
Symptomatic ICH within 24 h *n* (%)^?!^	0.26 (0.03 to 2.09)	0.204	0.06 (0.00 to 1.66)	0.096	1.46 (0.46 to 4.61)	0.524	1.48 (0.34 to 6.43)	0.602	0.58 (0.19 to 1.80)	0.344	0.43 (0.10 to 1.95)	0.275
Any ICH within 24 h, *n* (%)^??^	0.91 (0.45 to 1.87)	0.806	1.01 (0.46 to 2.23)	0.975	0.64 (0.29 to 1.44)	0.281	0.57 (0.24 to 1.36)	0.204	0.82 (0.46 to 1.45)	0.497	0.83 (0.41 to 1.67)	0.601
Death within 90 d, *n* (%)	1.11 (0.56 to 2.20)	0.774	1.38 (0.59 to 3.21)	0.452	0.43 (0.17 to 1.08)	0.074	0.74 (0.26 to 2.08)	0.563	0.80 (0.45 to 1.43)	0.459	1.32 (0.60 to 2.90)	0.493
Intraprocedural embolization, *n* (%)	0.59 (0.12 to 2.91)	0.516	0.89 (0.15 to 5.16)	0.898	0.65 (0.13 to 3.20)	0.594	0.38 (0.04 to 3.58)	0.400	1.06 (0.38 to 3.000)	0.907	1.48 (0.37 to 5.95)	0.583

OR, odds ratio; IA, intra-arterial; IV, intravenous; mRS, modified Rankin Scale; IQR, inter quartile range; NIHSS, National Institutes of Health Stroke Scale; ICH, intracranial hemorrhage.

* Adjusting for confounders including Male, Hypertension, Hyperlipidemia, Atrial fibrillation, Smoking history, admission systolic blood pressure in admission, admission ASPECTS, Occlusion sites, onset-to-door time, door to-puncture time, onset-to-recanalization time, onset-to-puncture time, Prior use of antiplatelet agents, IAT, and Rescue balloon/stenting angioplasty.

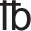
 31 missing data, the β-coefficients were calculated using a generalized linear model.

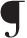
 39 missing data, the β-coefficients were calculated using a generalized linear model.

^??^13 missing data.

^?!^15 missing data.

In the analysis of postmatched outcomes, the common odds ratio (OR) for 90-day ordinal modified Rankin Scale distribution with IA-tirofiban vs. non-tirofiban was 0.41 (95% CI, 0.18–0.94, *P* = 0.036), and the shift on the 90-d mRS score in postmatched patients of non-tirofiban, IA-tirofiban, IV-tirofiban, and (IA+IV)-tirofiban groups was depicted in Supplementary Figure 1. The OR for mRS 0–1 and mRS 0–2 at 90-day with IA-tirofiban vs. non-tirofiban was, respectively 0.28 (95% CI, 0.11–0.74, *P* = 0.011) and 0.25 (95% CI, 0.09–0.67, *P* = 0.006; Supplementary Tables 2, 3).

### Subgroup analysis

As shown in Table 4, there were significant interaction effects between administration pathway of tirofiban and onset-to-puncture time (*p* for interaction = 0.028) and rescue balloon/stenting angioplasty (*P* for interaction = 0.008) on the 90-day mRS score. Intra-arterial tirofiban tended to be associated with worse outcome of 90-day mRS (adjusted common OR, 0.46; 95% CI, 0.21–1.00)in patients with onset-to-puncture time more than or equal 6 h, whereas not (adjusted common OR, 1.01; 95% CI, 0.45–2.27) in patients with onset-to-puncture time <6 h; intravenous tirofiban was associated with better outcome of 90-day mRS (adjusted common OR, 3.64; 95% CI, 1.57–8.43) in patients without receiving rescue balloon/stenting angioplasty, whereas not (adjusted common OR, 0.55; 95% CI, 0.25–1.22) in patients with receiving rescue balloon/stenting angioplasty. However, no interaction effect was found in other subgroups (all *P* for interaction >0.05; Table 4).

**Table 4:** Subgroup analysis regarding 90-day mRS of different tirofiban groups.

**Subgroup**	IA-tirofiban vs. non-tirofiban^*^	IV-tirofiban vs. non-tirofiban^*^	(IA+IV)- tirofiban vs. non-tirofiban^*^	***P* for interaction**
**Gender**				
Female	0.34 (0.09–1.39)	0.80 (0.27–2.40)	1.19 (0.40–3.54)	0.840
Male	0.72 (0.40–1.30)	1.48 (0.77–2.86)	0.99 (0.58–1.69)	
**Age**				
< 65	0.66 (0.33–1.35)	1.09 (0.52–2.30)	1.09 (0.57–2.07)	0.512
≥65	0.82 (0.36–1.87)	1.68 (0.71–3.97)	0.83 (0.41–1.70)	
**Baseline NIHSS**				
NIHSS < 15	1.06 (0.47–2.37)	1.35 (0.59–3.05)	0.86 (0.44–1.67)	0.707
NIHSS ≥ 15	0.65 (0.31–1.37)	1.38 (0.63–3.06)	1.11 (0.55–2.25)	
**Baseline ASPECTS**				
ASPECT ≤ 9	1.08 (0.53–2.18)	1.14 (0.54–2.38)	1.35 (0.75–2.45)	0.134
ASPECT = 10	0.61 (0.26–1.43)	1.62 (0.68–3.85)	0.58 (0.25–1.31)	
**Occlusion location**				
Posterior circulation	0.48 (0.17–1.34)	0.95 (0.26–3.52)	0.90 (0.34–2.37)	0.805
Anterior circulation	0.79 (0.41–1.52)	1.45 (0.77–2.71)	1.11 (0.64–1.93)	
**Tandem lesions**				
Yes	1.65 (0.43–6.32)	2.14 (0.59–7.84)	3.37 (1.12–10.08)	0.885
No	0.69 (0.38–1.27)	1.26 (0.66–2.39)	0.91 (0.53–1.57)	
**Onset–to–puncture time**				
< 6 h	1.01 (0.45–2.27)	1.32 (0.60–2.90)	0.60 (0.30–1.19)	0.028
≥6 h	0.46 (0.21–1.00)	1.40 (0.61–3.22)	1.40 (0.70–2.83)	
**Pretreatment with IVT**				
Yes	0.61 (0.20–1.83)	0.74 (0.20–2.65)	0.80 (0.30–2.12)	0.824
No	0.82 (0.44–1.55)	1.59 (0.85–2.99)	1.07 (0.61–1.86)	
**Rescue balloon/stenting angioplasty**				
Yes	0.51 (0.23–1.14)	0.55 (0.25–1.22)	0.89 (0.47–1.66)	0.008
No	0.97 (0.46–2.04)	3.64 (1.57–8.43)	0.82 (0.38–1.76)	

IA, intra-arterial; IV, intravenous; NIHSS, National Institutes of Health Stroke Scale; ASPECTS, Alberta Stroke Program Early CT Score; IVT, intravenous thrombolysis.

* Adjusting for confounders including Male, Hypertension, Hyperlipidemia, Atrial fibrillation, Smoking history, admission systolic blood pressure in admission, admission ASPECTS, Occlusion sites, onset-to-door time, door to-puncture time, onset-to-recanalization time, onset-to-puncture time, Prior use of antiplatelet agents, IAT, and Rescue balloon/stenting angioplasty.

## Discussion

In our study, we focused on patients with ICAD related LVO undergoing EVT, and we didn't find IA-tirofiban, IV-tirofiban, and (IA+IV) could significantly improve the primary outcome of the 90-day ordinal mRS. However, we found IA-tirofiban decreased the rates of mRS 0–1 and mRS 0–2 at 90 d in both the prematched and postmatched population. After PSM, we found that IA-tirofiban was associated with more severe disability of the 90-day ordinal mRS. Furthermore, in subgroup analysis we found IA-tirofiban tended to be harm to the primary outcome of the 90-day ordinal mRS in patients with onset-to-puncture time more than or equal 6 h and IV-tirofiban could improve the primary outcome of the 90-day ordinal mRS in patients without rescue balloon/stenting angioplasty.

Our study failed to find IV-tirofiban could improve prognosis of patients with ICAD related LVO, which isn't consistent with the subgroup analysis of the RESCUE BT trial (2). The following reasons were considered: (1) Our study was observational, although a multivariate logistic regression analysis was conducted, selective bias still existed in intravenous tirofiban and non-tirofiban groups. (2) Because effect size was small, our sample size couldn't detect difference. (3) The dose of tirofiban in our study was much lower than RESCUE BT. Infusion median speed of tirofiban in our study was 0.07 μg/kg/min, but in RESCUE BT was 0.15 μg/kg/min. In addition, we didn't find (IV+IA)-tirofiban was sufficient to improve prognostic outcomes. Even though in initial univariable analysis, the rate of complete recanalization was the highest in (IV+IA) group, (IV+IA)-tirofiban neither associated with complete recanalization nor successful recanalization after adjusting potential confounders. This finding was supported by a recent pool-analysis (4), and was inconsistent with previous study (13, 14). Given that our studies were non-randomized controlled, further research will be needed to verify.

Although the risk of symptomatic ICH was not significantly different between the three tirofiban groups compared with the non-tirofiban group, we found IA-tirofiban could decreased the rates of mRS 0–1 and mRS 0–2 at 90 d in patients with ICAD-related LVO undergoing EVT. We speculated that there were several reasons for this. First, intra-arterial injection increases the local drug concentration of tirofiban which might aggravate damage to the blood-brain barrier and lead to intracerebral hemorrhage due to the presence of ischemic brain tissue. This viewpoint is supported by two studies (3, 15), Wu et al. reported intra-arterial tirofiban administration increases risk of major ICH after endovascular thrombectomy for acute ischemic stroke, and doses more than 6.7 μ g/kg were associated with symptomatic and fatal intracranial hemorrhage (3). In our study, median bolus dose of IA-tirofiban was 5.7 μg/kg. Secondly, administration of IA-tirofiban before EVT might increase the risk of thrombus migration toward distal blood vessel, because the clot in ICAD-LVO is fresh and intra-arterial injection allows tirofiban directly contacts with the thrombus and might promote thrombus evolution. It was reported that IV-tPA (Tissue-Type Plasminogen Activator) administration before EVT for LVO was associated with distal embolization, which in turn might reduce the chance that recanalization was achieved (16), so we speculated that IA-tirofiban had the same effect. Thirdly, arterial injection of tirofiban alone is not a continuous dose which might have a poor preventive effect on late *in situ* thrombosis and reocclusion (17–19). However, some study showed contrary conclusion that intra-arterial tirofiban didn't increase risk of ICH, even improved the clinical outcome of patients undergoing EVT (20). Up to now, there has been no randomized controlled trial about intra-arterial tirofiban administration in patients undergoing EVT. All studies were based on real world observational study. The safety of intra-arterial tirofiban treatment in patients with ICAD related LVO requires further randomized controlled trial to be verified. Therefore, intra-arterial tirofiban should be administrated cautiously during EVT, if necessary, a low dose may be more feasible.

In subgroup analyses, we found intra-arterial tirofiban tended to be associated with worse outcome of 90-day mRS in patient with onset-to-puncture time more than or equal 360 min. Possible explanation for this finding was that infarct grew, and vascular bed was destroyed more widely with a longer onset-to-puncture time, so IA-tirofiban administration increased risk of ICH. We also found intravenous tirofiban was associated with better outcome of 90-day mRS in patients without receiving rescue balloon/stenting angioplasty. It was reported that re-occlusion after an initial recanalization with SR thrombectomy in ICAS-related LVO was very frequent (65%) (21), the main causes for re-occlusion are residual plaque and platelet activation leading to thrombosis (22). If patients with ICAS-related LVO don't receive rescue balloon/stenting angioplasty, we believe that early and long-lasting intravenous tirofiban is preventive for the re-occlusion.

Our study has several limitations. First, this was not a randomized control trial, patients didn't have equal chance to enter each group, and measured and unmeasured variables still acted on the effect size, although we conducted a logistic regression to adjust for confounders. Second, there was no unified mandatory regime for tirofiban, the use of tirofiban was finally at the discretion of the treating physician and local practice in the present study, and different dose and period of the procedure may lead to different endpoint events. Third, we did not analyze the status of the perfusion, collateral, social background, economic situation, and genes of patients which are important factors for a good prognosis. Fourth, in our study underlying ICAD was defined as fixed stenosis degree >70% or stenosis >50% with distal blood flow impairment or evidence of repeated re-occlusion, this definition might mistake residual thrombus after thrombectomy as an intracranial atherosclerotic stenosis lesion. Last, our study population was limited to the Chinese population, which confined the generalizability of our results.

## Conclusion

Administration of IA-tirofiban had a harm effect on patients undergoing EVT for ICAD-related LVO, especially in patients with onset-to-puncture time more than or equal 6 h instead of increasing the rates of complete recanalization and successful recanalization compared with non-tirofiban; administration of intravenous tirofiban could improve the prognosis of patients undergoing EVT for ICAD-related LVO without receiving rescue balloon/stenting angioplasty.

## Data availability statement

The raw data supporting the conclusions of this article will be made available by the authors, without undue reservation.

## Ethics statement

The studies involving humans were approved by the Ethics Committees of Beijing Tiantan Hospital, Capital Medical University. The studies were conducted in accordance with the local legislation and institutional requirements. Written informed consent for participation was not required from the participants or the participants' legal guardians/next of kin in accordance with the national legislation and institutional requirements.

## Author contributions

ZB: Writing—original draft, Conceptualization, Data curation, Formal analysis, Investigation, Methodology, Resources, Validation, Visualization, Writing—review & editing. DSun: Conceptualization, Data curation, Formal analysis, Methodology, Resources, Validation, Visualization, Writing—review & editing. GM: Conceptualization, Writing—review & editing. BJ: Conceptualization, Data curation, Writing—review & editing. XT: Writing—review & editing. XH: Writing—review & editing. AW: Software, Writing—review & editing. NM: Writing—review & editing. FG: Writing—review & editing. DM: Writing—review & editing. LS: Writing—review & editing. XS: Writing—review & editing. YD: Writing—review & editing. XL: Writing—review & editing. BW: Writing—review & editing. GL: Writing—review & editing. DSu: Supervision, Writing—review & editing. ZM: Conceptualization, Funding acquisition, Methodology, Project administration, Resources, Supervision, Visualization, Writing—review & editing.
