# Supplementary material for: The impact of intraarterial, intravenous, and combined tirofiban on endovascular treatment for acute intracranial atherosclerotic occlusion
Source: Front Neurol. 2024 Feb 13;15:1336098. doi: 10.3389/fneur.2024.1336098 (PMC10896834; doi:10.3389/fneur.2024.1336098)
Supplement: Supplementary file 1 [file Data_Sheet_1.docx]

**Data Supplement**

**Table I Baseline Characteristics of Different Tirofiban Groups in Postmatched Population**

| Variables | Non-tirofiban (*n* = 36) | IA-tirofiban (n = 36) | IV-tirofiban (n = 36) | (IA+IV)-tirofiban (n = 36) | Standardized difference, % | P-value |
| --- | --- | --- | --- | --- | --- | --- |
| Baseline characteristics | | | | | | |
| Male, n (%) | 27(75.0) | 30(83.3) | 29(80.6) | 30(83.3) | -0.21 | 0.786 |
| Age, y, median (IQR) | 60(52.5-66.5) | 60(54.5-70) | 60(51.5-66.5) | 63.5(55.0-71.0) | 0.18 | 0.542 |
| Hypertension, n (%) | 23(63.9) | 20(55.6) | 20(55.6) | 20(55.6) | -0.17 | 0.857 |
| Diabetes, n (%) | 5(13.9) | 12(33.3) | 7(19.4) | 9(25.0) | 0.47 | 0.240 |
| Hyperlipidemia, n (% | 2(5.6) | 0(0.0) | 4(11.1) | 1(2.8) | -0.34 | 0.154 |
| Coronary heart disease, n (%) | 3(8.3) | 3(8.3) | 3(8.3) | 3(8.3) | 0.00 | 1.000 |
| Atrial fibrillation, n (%) | 2(5.6) | 5(13.9) | 2(5.6) | 2(5.6) | 0.28 | 0.448 |
| Prior stroke, n (%) | 3(8.3) | 7(19.4) | 7(19.4) | 9(25.0) | 0.33 | 0.312 |
| Smoking history, n (%) |  |  |  |  |  |  |
| Never smoking | 16(44.4) | 21(58.3) | 14(38.9) | 14(38.9) | 0.35 | 0.588 |
| Current smoking | 18(50.0) | 12(33.3) | 19(52.8) | 20(55.6) |  |  |
| Previous smoking | 2(5.6) | 3(8.3) | 3(8.3) | 2(5.6) |  |  |
| SBP, mmHg, median (IQR) | 150(130.5-166) | 145(130-165.5) | 145.5(138.5-160) | 145.5(134.5-167.5) | -0.07 | 0.945 |
| Admission NIHSS, median (IQR) | 15(11-19.5) | 14(9-22) | 15(9-18.5) | 15(12.5-21.5) | 0.00 | 0.695 |
| Admission ASPECTS ,median (IQR) | 9(7-10) | 9(8-10) | 9.5(7-10) | 8(7-10) | 0.21 | 0.485 |
| Anterior circulation | 28(77.8) | 26(72.2) | 29(80.6) | 27(75.0) | -0.13 | 0.857 |
| Posterior circulation | 8(22.2) | 10(27.8) | 7(19.4) | 9(25.0) | 0.13 | 0.857 |
| Occlusion sites | | | | | | |
| ICA | 5(13.9) | 5(13.9) | 8(22.2) | 3(8.3) | 0.26 | 0.855 |
| M1 | 21(58.3) | 17(47.2) | 19(52.8) | 22(61.1) |  |  |
| VBA | 8(22.2) | 10(27.8) | 7(19.4) | 8(22.2) |  |  |
| Other^℥^ | 2(5.6) | 4(11.1) | 2(5.6) | 3(8.3) |  |  |
| Tandem lesions | 11(30.6) | 9(25.0) | 9(25.0) | 8(22.2) | -0.12 | 0.875 |
| OTD time, median (IQR), min | 181.5(78.5-447.5) | 220.5(123.5-324.5) | 187.5(113-315) | 252.5(157.5-349.5) | -0.06 | 0.812 |
| DTP time, median (IQR), min | 99(69-136.5) | 150.5(75-265.5) | 111.5(65.5-164.5) | 110(78-151) | 0.52 | 0.189 |
| PTR time, median (IQR), min | 108.5(65-142.5) | 106.5(78-176.5) | 107.5(69.5-149) | 80.5(48-175) | 0.19 | 0.760 |
| OTR time, median (IQR), min | 428(370.5-597.5) | 521(436-657) | 472(302.5-623.5) | 481.5(359-602) | 0.27 | 0.255 |
| OTP time, median (IQR), min | 344.5(230-518) | 375(279.5-539.0) | 334(216.5-490) | 362.5(265-472.5) | 0.21 | 0.502 |
| General anesthesia, n (%) | 17(47.2) | 15(41.7) | 17(47.2) | 13(36.1) | -0.11 | 0.742 |
| Prior use of antiplatelet agents | 3(8.3) | 5(13.9) | 4(11.1) | 4(11.1) | 0.18 | 0.905 |
| Prior use of anticoagulants | 0(0.0) | 1(2.8) | 0(0.0) | 0(0.0) | 0.24 | 0.388 |
| Prior IVT, n (%) | 11(30.6) | 12(33.3) | 6(16.7) | 11(30.6) | 0.06 | 0.385 |
| Heparin, n (%) | 19(52.8) | 18(50.0) | 15(41.7) | 18(50.0) | -0.06 | 0.801 |
| IAT, n (%) | 2(5.6) | 4(11.1) | 2(5.6) | 2(5.6) | 0.20 | 0.732 |
| Stent retriever as first-line, n (%) | 20(55.6) | 24(66.7) | 20(55.6) | 25(69.4) | 0.23 | 0.486 |
| Direct aspiration as first-line, n (%) | 1(2.8) | 2(5.6) | 0(0.0) | 3(8.3) | 0.14 | 0.324 |
| Direct aspiration+ stent retriever as first-line | 5(13.9) | 2(5.6) | 5(13.9) | 2(5.6) | -0.28 | 0.416 |
| Rescue balloon/stenting angioplasty, n (%) | 20(55.6) | 13(36.1) | 18(50.0) | 20(55.6) | -0.40 | 0.303 |

**Abbreviations:** IA, intra-arterial; IV, intravenous; SBP, systolic blood pressure; IQR, interquartile range; NIHSS, National Institutes of Health Stroke Scale; ASPECTS, Alberta Stroke Program Early CT Score; ICA, internal carotid artery; M1, middle cerebral artery M1 segment; VBA, Vertebrobasilar artery; OTD, onset-to-door; DTP, door to-puncture; PTR, puncture-to-recanalization; OTR, onset-to-recanalization; OTP, onset-to-puncture; IVT, intravenous thrombolysis; IAT, intra-arterial thrombolysis.

℥ Other including middle cerebral artery M2 segment, anterior cerebral artery A1/A2 segments and posterior cerebral artery P1 segment.

**Table II Outcomes of Different Tirofiban Groups in Postmatched Population**

| Variables | Non-tirofiban (*n* = 36) | IA-tirofiban (n = 36) | IV-tirofiban (n = 36) | (IA+IV)- tirofiban (n = 36) | P-value |
| --- | --- | --- | --- | --- | --- |
| mRS at 90 d, median (IQR) | 1(0-4) | 3.5(1-5) | 2(0-4) | 3(0.5-4) | 0.126 |
| mRS 0–1 at 90 d, n (%) | 22(61.1) | 11(30.6) | 17(47.2) | 15(41.7) | 0.071 |
| mRS 0–2 at 90 d, n (%) | 23(63.9) | 11(30.6) | 19(52.8) | 15(41.7) | 0.030 |
| mRS 0–3 at 90 d, n (%) | 25(69.4) | 18(50.0) | 22(61.1) | 19(52.8) | 0.330 |
| Change in NIHSS score at 24 h, median (IQR) ^℔^ | -4(-7 to -1) | -1.5(-6 to 1) | -3(-8 to -1) | -4(-9 to -1) | 0.352 |
| Change in NIHSS score at 7 d, median (IQR)^❡^ | -6(-11 to -3) | -4(-9 to -1) | -7(-11.5 to -3) | -7(-12 to -1) | 0.499 |
| complete recanalization , n (%) | 19(52.8) | 18(50.0) | 19(52.8) | 21(58.3) | 0.912 |
| successful recanalization, n (%) | 34(94.4) | 30(83.3) | 31(86.1) | 33(91.7) | 0.421 |
| Pass number of thrombectomy, median (IQR) | 2(1-2) | 1.5(1-3) | 1(1-2) | 1(1-2) | 0.634 |
| Symptomatic ICH within 24 h n (%)^⁈^ | 0(0.0) | 1(2.8) | 3(8.3) | 2(5.7) | 0.322 |
| Any ICH within 24 h, n (%)^⁇^ | 6(16.7) | 10(27.8) | 6(16.7) | 7(20.0) | 0.607 |
| Death within 90 d, n (%) | 5(13.9) | 5(13.9) | 3(8.3) | 6(16.7) | 0.765 |
| Intraprocedural embolization, n (%) | 2(5.6) | 0(0.0) | 0(0.0) | 2(5.6) | 0.249 |

**Abbreviations:** IA, intra-arterial; IV, intravenous; mRS modified Rankin Scale; IQR, interquartile range; NIHSS, National Institutes of Health Stroke Scale; ICH intracranial hemorrhage.

℔ 8 missing data in postmatched population; ❡ 11 missing data in postmatched population; ⁇ 1missing data in postmatched population; ⁈ 1 missing data in postmatched population.

**Table III**

**Common OR or OR of Safety and Efficacy Outcome According to Different Regimen of Tirofiban in Postmatched Population**

| Outcomes Variables | IA-tirofiban vs non-tirofiban | | IV-tirofiban vs non-tirofiban | | (IA+IV)- tirofiban vs non-tirofiban | |
| --- | --- | --- | --- | --- | --- | --- |
|  | Effect size, (95%CI) | P-value | Effect size, (95%CI) | P-value | Effect size, (95%CI) | P-value |
| mRS at 90 d, median (IQR) | 0.41(0.18-0.94) | 0.036 | 0.85(0.37-1.95) | 0.696 | 0.52(0.23-1.20) | 0.124 |
| mRS 0–1 at 90 d, n (%) | 0.28(0.11-0.74) | 0.011 | 0.57(0.22-1.45) | 0.239 | 0.46(0.18-1.17) | 0.101 |
| mRS 0–2 at 90 d, n (%) | 0.25(0.09-0.67) | 0.006 | 0.63(0.25-1.62) | 0.340 | 0.40(0.16-1.04) | 0.061 |
| mRS 0–3 at 90 d, n (%) | 0.44(0.17-1.16) | 0.095 | 0.69(0.26-1.84) | 0.459 | 0.49(0.19-1.29) | 0.150 |
| Change in NIHSS score at 24 h, median (IQR)℔ | 1.43(-2.50 to 5.37) | 0.472 | 3.26(-0.68 to 7.19) | 0.104 | 0.98(-2.98 to 4.95) | 0.624 |
| Change in NIHSS score at 7 d, median (IQR)^❡^ | 1.59(-2.39 to 5.57) | 0.432 | 3.60(-0.41 to 7.61) | 0.078 | 0.99(-3.06 to 5.03) | 0.629 |
| complete recanalization , n (%) | 0.90(0.36-2.26) | 0.814 | 1.00(0.40-2.52) | 1.000 | 1.25(0.49-3.18) | 0.635 |
| successful recanalization, n (%) | 0.29(0.06-1.57) | 0.152 | 0.37(0.07-2.02) | 0.248 | 0.65(0.10 -4.12) | 0.645 |
| Pass number of thrombectomy, median (IQR) | 0.36(-0.31 to 1.04) | 0.292 | 0.53(-0.15 to 1.20) | 0.125 | 0.17(-0.51 to 0.84) | 0.626 |
| Symptomatic ICH within 24 h n (%)^⁈^ | ― | ― | ― | ― | ― | ― |
| Any ICH within 24 h, n (%)^⁇^ | 1.92(0.62-6.02) | 0.261 | 1.00(0.29-3.45) | 1.000 | 1.25(0.37-4.18) | 0.717 |
| Death within 90 d, n (%) | 1.00(0.26-3.80) | 1.000 | 0.56(0.12-2.56) | 0.458 | 1.24(0.34-4.50) | 0.744 |
| Intraprocedural embolization, n (%) | ― | ― | ― | ― | 1.00(0.13-7.51) | 1.000 |

**Abbreviations:** OR, odds ratio; IA, intra-arterial; IV, intravenous; mRS modified Rankin Scale; IQR, interq uartile range; NIHSS, National Institutes of Health Stroke Scale; ICH intracranial hemorrhage.

℔ 8 missing data in postmatched population; ❡ 11 missing data in postmatched population; ⁇ 1missing data in postmatched population; ⁈ 1 missing data in postmatched population.

**
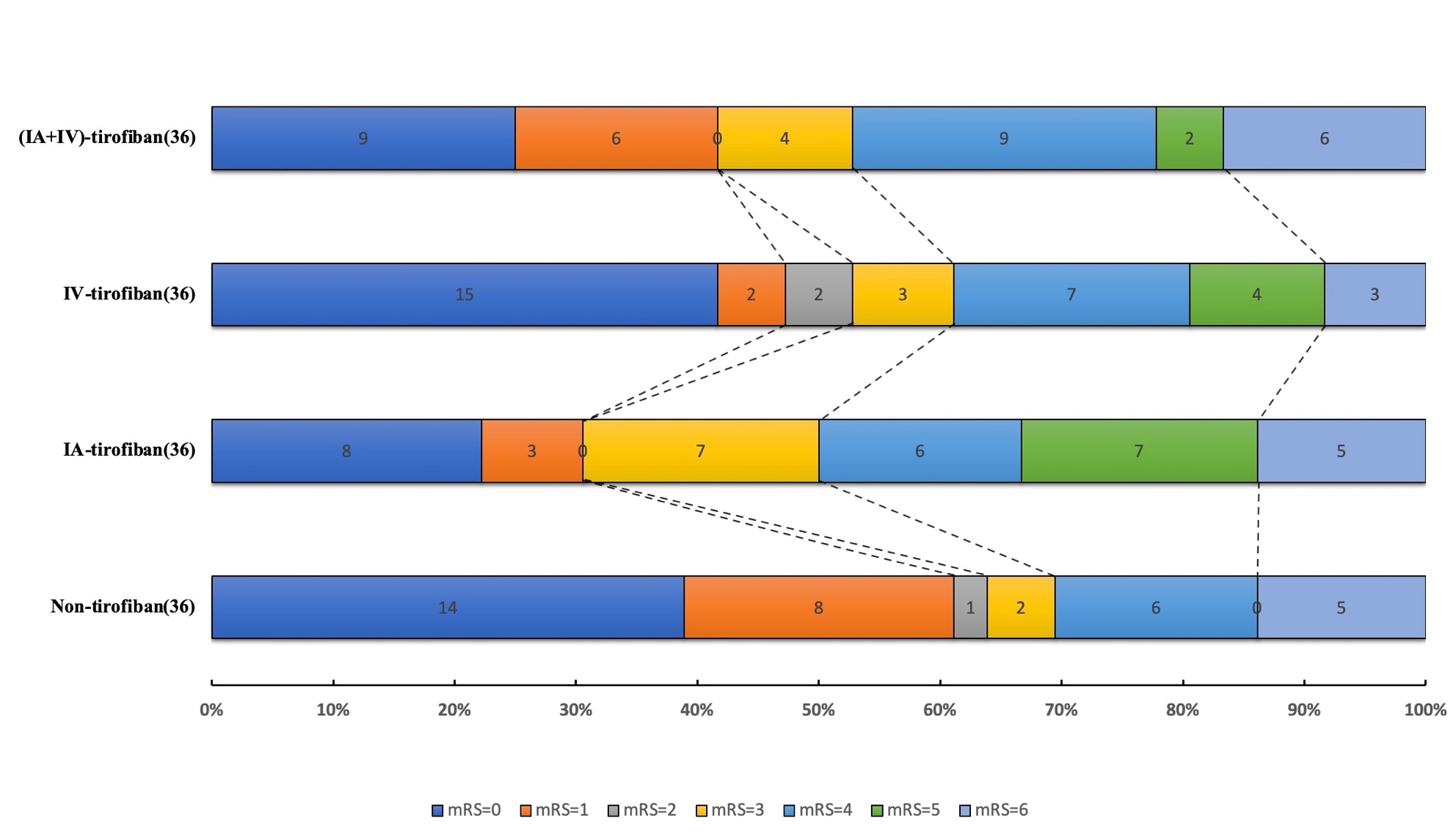
**

**Figure I The shift on the 90-d modified Rankin Scale (mRS) score in postmatched patients of non-tirofiban, intra-arterial (IA) tirofiban, intravenous (IV) tirofiban and intra-arterial plus intravenous (IA+IV) tirofiban.**
